# Estimation of the synaptic input firing rates and characterization of the stimulation effects in an auditory neuron

**DOI:** 10.3389/fncom.2015.00059

**Published:** 2015-05-18

**Authors:** Ryota Kobayashi, Jufang He, Petr Lansky

**Affiliations:** ^1^Principles of Informatics Research Division, National Institute of InformaticsTokyo, Japan; ^2^Department of Informatics, SOKENDAI (The Graduate University for Advanced Studies)Tokyo, Japan; ^3^Department of Biomedical Sciences, City University of Hong KongHong Kong, China; ^4^Institute of Physiology, The Czech Academy of SciencesPrague, Czech Republic

**Keywords:** synaptic inputs, statistical inference, state-space models, intracellular recordings, auditory cortex

## Abstract

To understand information processing in neuronal circuits, it is important to infer how a sensory stimulus impacts on the synaptic input to a neuron. An increase in neuronal firing during the stimulation results from pure excitation or from a combination of excitation and inhibition. Here, we develop a method for estimating the rates of the excitatory and inhibitory synaptic inputs from a membrane voltage trace of a neuron. The method is based on a modified Ornstein-Uhlenbeck neuronal model, which aims to describe the stimulation effects on the synaptic input. The method is tested using a single-compartment neuron model with a realistic description of synaptic inputs, and it is applied to an intracellular voltage trace recorded from an auditory neuron *in* *vivo*. We find that the excitatory and inhibitory inputs increase during stimulation, suggesting that the acoustic stimuli are encoded by a combination of excitation and inhibition.

## Introduction

Cortical neurons *in vivo* exhibit irregular firing patterns even in a high firing regime (Softky and Koch, 1993). This irregular firing pattern may be explained by synaptic inputs having balanced excitation and inhibition (Shadlen and Newsome, 1998), which causes the membrane potential to fluctuate predominantly below the spike threshold. Thus, the neuron randomly generates action potentials (spikes). To assess the hypothesis, it is essential to determine the synaptic inputs to the neuron. Because their direct measurement is beyond the capacity of current technology, attempts to deduce them have been based on experimentally measurable quantities, such as membrane voltage (Lansky, 1983; Rudolph et al., 2004; Lansky et al., 2006, 2010; Kobayashi et al., 2011a,b; Bedard et al., 2012; Paninski et al., 2012; Berg and Ditlevsen, 2013; Lankarany et al., 2013), voltage-clamp data (Borg-Graham et al., 1998; Wehr and Zador, 2003) or spike trains (Shinomoto et al., 1999; Ditlevsen and Lansky, 2005; Kim and Shinomoto, 2012). These attempts have two equally important components. The first component is to construct a mathematical model that relates the synaptic inputs to the available experimental data. The second component is to devise a method for estimating the input from these data.

The leaky integrate-and-fire (LIF) concept is widely used in computational neuroscience (e.g., Tuckwell, 1988; Gerstner and Kistler, 2002). It assumes that the voltage is described by an RC circuit, which consists of a capacitor and a resistor in parallel. Despite the simplicity of the LIF model and its generalizations, they provide a good approximation of single-compartment conductance-based models, including the Hodgkin—Huxley model (Abbott and Kepler, 1990; Destexhe, 1997; Gerstner and Kistler, 2002; Jolivet et al., 2004; Kobayashi and Shinomoto, 2007). A generalized LIF model is used here for direct interpretation of the measurable variable, i.e., the membrane depolarization, in terms of the neuronal input represented by the activity of the excitatory and inhibitory presynaptic neurons.

The LIF model with Gaussian white noise input, which is referred to as the Ornstein—Uhlenbeck (OU) model, has often been considered for the purpose of inference regarding the input. Abstract quantities, such as the mean and variance of synaptic currents, and the synaptic input rates, which are defined as the number of input spikes from pre-synaptic neurons per unit time, were estimated based on the OU model (Lansky, 1983; Shinomoto et al., 1999; Lansky et al., 2006, 2010; Kobayashi et al., 2011a). Physiological quantities, i.e., synaptic conductance, were estimated using a modified OU model (Rudolph et al., 2004) or a deterministic LIF model (Borg-Graham et al., 1998; Wehr and Zador, 2003; Berg et al., 2007; for a review, see Monier et al., 2008). Recently, these methods were extended to identify the input variations from a single voltage trace, which are relatively easy to obtain experimentally (Kobayashi et al., 2011a,b; Bedard et al., 2012; Paninski et al., 2012; Berg and Ditlevsen, 2013; Lankarany et al., 2013).

Here, we develop a method to characterize the effect of stimulation on synaptic input from a single voltage trace. We examine whether the method can characterize the effect of stimulation on the synaptic input. After verification with simulated data, we apply the method to *in vivo* recordings (He, 2003; Lansky et al., 2006, 2010) and investigate the effect of stimulation on an auditory thalamic neuron.

## Materials and methods

We first summarize the basic properties of the OU model with afterhyperpolarization (AHP), which is used to estimate the input signals. The estimation method is described in Section Estimation of Input Signals, and a neuron model that is used to generate synthetic data is introduced in Section Single-compartment Neuron Model with Realistic Synaptic Inputs. Finally, we briefly describe how the experimental data were collected and processed.

### OU model with AHP and characterization of the stimulus effect

The subthreshold voltage V(t) of a neuron is decomposed into two parts,

(1) $$V(t)=U(t)+h\left(t-t_{f}\right),$$

where *t*_*f*_ is the time of the most recent spike occurrence, *h*(*t*) reflects the AHP, and *U*(*t*) is the OU stochastic process describing the depolarization in the absence of spiking,

(2) $$\frac{dU}{dt}=-\frac{U(t)-u_{0}}{\tau_{m}}+\mu (t)+\sigma (t)\xi (t)$$

where *u*_0_ is the resting potential, τ_*m*_ is the membrane time constant, μ(*t*) is the input mean, σ^2^(*t*) is the input variance, and ξ (*t*) is a Gaussian white noise with zero mean and unit standard deviation (SD). The initial value of *U*(*t*) plays no role because the process continues without being influenced by the spikes. Throughout the paper, only for notational convenience, we refer to *U*(*t*) as the potential to distinguish this term from the voltage *V*(*t*) given by Equation (1).

**Figure 1:**
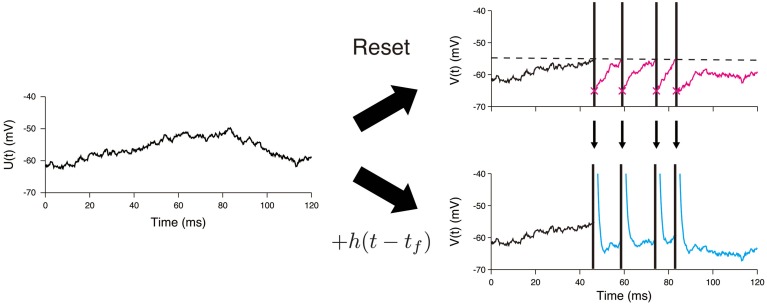
**Difference between the LIF model and the OU-AHP model**. A sample path of the Ornstein—Uhlenbeck process is shown on the left panel. In the LIF model, the voltage is reset just after it reaches the threshold (dashed line) (Top). On the other hand, the spike times are necessary to calculate the voltage trace in the OU-AHP model (Bottom). The AHP function *h*(*t*) added to the potential *U*(*t*).

The main difference between the OU-AHP model (1, 2) and the LIF model with white noise input is in the description of the spike-after effect (Figure 1). This effect is modeled via a simple instantaneous reset in the LIF model (Tuckwell, 1988; Lansky and Ditlevsen, 2008), whereas it is described by the addition of the AHP function *h*(*t*) in the OU-AHP model. It should be noted that the OU-AHP model, which is similar to the model considered in Lansky et al. (1992) is a special case of the spike response model (Gerstner and Kistler, 2002) with white noise input. As indicated in the literature (Gerstner and Kistler, 2002; Jolivet et al., 2004; Kobayashi and Shinomoto, 2007), the spike response model accurately reproduces the membrane voltage of single-compartment Hodgkin-Huxley type models.

The input to the model neuron (1, 2) is determined by two functions: μ(*t*) and σ^2^(*t*), which we refer to as input statistics. For stationary input, μ(*t*) = μ_0_, and σ^2^(*t*) = σ^2^_0_, Equation (2) is characterized by the asymptotic mean and variance:

(3) $$E\left[U\left(\infty \right)\right]=\mu_{0}\tau_{m}+u_{0},\;Var\left[U\left(\infty \right)\right]=\;\sigma_{0}^{2}\;\tau_{m}/2.$$

Assuming that the spike trains of the presynaptic neurons can be approximated by Poisson process, the input statistics can be related to the amplitudes and the rates of the post-synaptic potentials by using the diffusion approximation (Tuckwell, 1988).

(4) $$\mu (t)=a_{E}\lambda_{E}(t)-a_{I}\lambda_{I}(t),\;\sigma^{2}(t)=\;a_{E}^{2}\lambda_{E}(t)+a_{I}^{2}\lambda_{I}(t).$$

where λ_*E*_ and λ_*I*_ are the total input rates from presynaptic neurons, *a*_*E*_ and *a*_*I*_ are the amplitudes of the post-synaptic potentials, and the indexes *E* and *I* represent the excitatory and inhibitory presynaptic neurons, respectively. The excitatory and inhibitory post-synaptic potential (EPSP and IPSP) are described by Dirac's delta function under the assumption that the synaptic time constants are small. Equation (4) is important because it relates abstract quantities (input statistics: μ, σ^2^) to physiologically relevant quantities (total firing rates of presynaptic neurons: λ_*E*_, λ_*I*_, and post-synaptic potentials: *a*_*E*_, *a*_*I*_). It should be noted that the OU-AHP model is a single-compartment model that describes the membrane voltage at the soma; therefore, the EPSP and IPSP refer to the contribution of synaptic inputs to the somatic voltage, not to the post-synaptic potentials in dendrites.

Within the described model, it is possible to interpret the stimulus effects in terms of the excitatory and inhibitory input rates, λ_*E*_ and λ_*I*_, respectively. The stimulus may or may not impact the synaptic inputs. Our aim is to determine whether there is an impact and if so, to deduce the effect. These detected effects can be excitatory or inhibitory. For a stimulus with an excitatory effect, the two scenarios, i.e., pure excitation and mixed excitation and inhibition, are possible. Pure excitation predominantly increases λ_*E*_, whereas mixed excitation and inhibition increases both λ_*E*_ and λ_*I*_.

### Estimation of input signals

**Figure 2:**
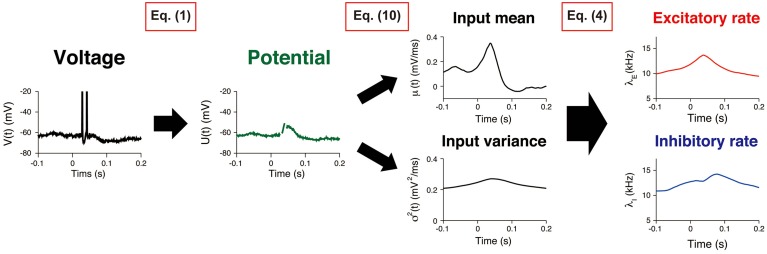
**Estimating synaptic input rates from a voltage trace**. A voltage trace is shown on the left panel. First, the potential U(*t*) is calculated by removing AHP (Equation 1). Then, the input mean and variance μ(t), σ^2^(*t*) are estimated by using the Kalman filtering technique (Equation 10). Finally, the excitatory and inhibitory input rates λ_*E*_(*t*), λ_*I*_(*t*) are deduced by using Equation (4).

The procedure comprised three consecutive steps (Figure 2). First, the potential *U*(*t*) was calculated from the recorded voltage trace of a neuron. The input statistics (μ(*t*), σ^2^(*t*)) were subsequently estimated, and the synaptic input rates (λ_*E*_(*t*), λ_*I*_(*t*)) were evaluated.

For the evaluation of the potential *U*(*t*), it is necessary to identify the AHP function *h*(*t*), which was determined by the least-square method. Given the assumption that the input signals are constant, the mean voltage was obtained by calculating the mean of Equation (1):

(5) $$E[V(t)]=E[U\left(\infty \right)]+h\left(t-t_{f}\right),$$

and the squared error ε^2^ was minimized with respect to *E*[*U*(∞)] and *h*(*t*),

(6) $$\varepsilon^{2}=\;\sum_{t}{\left\{V(t)-E\left[U\left(\infty \right)\right]-h\left(t-t_{f}\right)\right\}}^{2},$$

where the sum over all of the sampling times was calculated. No functional form was assigned to *h*(*t*); however it was assumed that it diminishes for large *t*-values, specifically, *h*(*t*) = 0 for *t* > 0.5 s. The potential *U*(*t*) was calculated by subtracting the AHP function *h*(*t*) from the voltage *V*(*t*).

The input statistics was estimated from the potential *U*(*t*). Let us assume that the voltage is sampled at *N* steps *t*_*j*_ (*j* = 1, …, *N*), whose sampling interval was given by Δ_*j*_ = *t*_*j* + 1_ − *t*_*j*_. Equation (2) was discretized as follows:

(7) $$U_{j+1}=U_{j}-\frac{\Delta_{j}}{\tau_{m}}\;\left(U_{j}-u_{0}\right)+M_{j}\Delta_{j}+e^{\frac{S_{t}}{2}}\sqrt{\Delta_{j}}\;\eta_{j}^{U},$$

where Δ_*j*_ is a sampling interval, η^*U*^_*j*_ are independent Gaussian random variables of zero mean and unit SD, and *U*_*j*_: = *U*(*t*_*j*_), *M*_*j*_: = μ(*t*_*j*_), and *e*^*S*_*t*_^: = σ^2^(*t*_*j*_) are discretized values. The membrane time constant, τ_*m*_, was determined from the auto-correlation function of the membrane voltage after spike removal (Berg and Ditlevsen, 2013). The exponential function was introduced in the last term of Equation (7) to ensure the strict positivity of the input variance (Smith et al., 2010), which was different from the previous algorithms (Kobayashi et al., 2011a,b). This modification improved the robustness of the algorithm (data not shown).

The random walk priors were assumed for *M*_*j*_ and *S*_*j*_ (Kitagawa and Gersch, 1996; Koyama and Shinomoto, 2005; Smith et al., 2010; Kobayashi et al., 2011a,b),

(8) $$M_{j+1}=M_{j}+\gamma_{M}\sqrt{\Delta_{j}}\;\eta_{j}^{M},\;S_{j+1}=S_{j}+\gamma_{S}\sqrt{\Delta_{j}}\;\eta_{j}^{S},$$

where γ_*M*_ and γ_*S*_ are the hyperparameters, and η^*M*^_*j*_ and η^*S*^_*j*_ are independent Gaussian random variables of zero mean and unit SD, respectively. The procedure to determine the hyperparameters is summarized in the Supplementary Material (Appendix A). One of the risks associated with the use of large hyperparameters is an over-fitting, i.e., the estimates could be contaminated by the random component of the data. To avoid this risk, the upper bounds of the hyperparameters were set to γ_*M*_ = 0.02 and γ_*S*_ = 0.01. Equations (7) and (8) can be written as the State-Space model:

(9) $${\vec{X}}_{j+1}={\vec{X}}_{j}+{\vec{\eta }}_{j}^{X},\;Z_{j}=M_{j}\Delta_{j}+e^{\frac{S_{t}}{2}}\sqrt{\Delta_{j}}\;\eta_{j}^{Z},$$

where ${\vec{X}}_{j}:=(M_{j},S_{j})$ are the state vectors, $Z_{j}:=U_{j+1}-U_{j}+\frac{\Delta_{j}}{\tau_{m}}\left(U_{j}-u_{0}\right)$ are the observations, ${\vec{\eta }}_{j}^{X}$ are the two-dimensional Gaussian random variables with zero mean and diagonal covariance matrix $G:=\text{diag}\left(\gamma_{M}^{2}{△}_{j},\gamma_{S}^{2}{△}_{j}\right)$, and η^*Z*^_*j*_ are independent Gaussian random variables of zero mean and unit SD. The observations *Z*_*j*_ were evaluated with the exception of the spike onsets. The input signals $\left\{M_{j},S_{j}\right\}$ were estimated by calculating the Bayesian estimators:

(10) $$\begin{array}{l} {\hat{M}}_{j}=E[M_{j}|Z_{1},Z_{2},\cdots ,\;Z_{N-1},\;\gamma_{M}],\\ \;{\hat{S}}_{j}=E[S_{j}|Z_{1},Z_{2},\cdots ,\;Z_{N-1},\;\gamma_{S}], \end{array}$$

where *N* is the length of voltage recording with a time step Δ_*j*_. The procedure to calculate the Bayesian estimators (10) is summarized in the Supplementary Material (Appendix B).

### Single-compartment neuron model with realistic synaptic inputs

A single-compartment neuron model with realistic synaptic inputs was used to stimulate the voltage trace of a neuron *in vivo* (Gerstner and Kistler, 2002). The membrane voltage *V*(*t*) was described as follows:

(11) $$C_{m}\frac{dV(t)}{dt}=-g_{L}\left(V(t)-E_{L}\right)+\frac{1}{a}I_{E}(t)+\frac{1}{a}I_{I}(t),$$

where *C*_*m*_ is the membrane capacitance, *g*_*L*_ is the leak conductance, *E*_*L*_ is the resting potential, *a* is the membrane area, and *I*_*E*_(*t*) and *I*_*I*_(*t*) are the excitatory and inhibitory synaptic currents, respectively. The leak conductance was set to *g*_*L*_ = 0.01 mS/cm^2^ to reproduce the experimental data. The other parameters were *C*_*m*_ = 1.0 μF/cm^2^, *E*_*L*_ = −70 mV and *a* = 3.5×10^4^ μm^2^, which were adopted from Destexhe et al. (1998). The synaptic currents were given by:

(12) $$\begin{array}{l} I_{E}(t)=-\sum_{k\;=\;1}^{N_{E}}g_{E,k}(t)(V-V_{E}),\\ I_{I}(t)\;=-\sum_{k\;=\;1}^{N_{I}}g_{I,k}(t)(V-V_{I}) \end{array}$$

where *N*_*E*_ and *N*_*I*_ are the numbers of presynaptic neurons, *g*_*E*, *k*_ and *g*_*I*, *k*_ are the synaptic conductances evoked by the k-th presynaptic neuron, and *V*_*E*_ and *V*_*I*_ are the reversal potentials. The synaptic conductances are described by a simple exponential decay with time constants τ_*E*_, τ_*I*_,

(13) $$g_{E,k}(t)={\bar{g}}_{E}e^{-(t-t_{f,k})/\tau_{E}},g_{I,k}(t)={\bar{g}}_{I}e^{-(t-t_{f,k})\text{/}\tau_{I}}$$

where *g*_*E*_ and *g*_*I*_ are the amplitudes of the post-synaptic conductance, and *t*_*f*, *k*_ is the most recent firing time of the k-th presynaptic neuron. The spike trains of the presynaptic neurons were generated by Poisson process.

There are two differences between the OU-AHP model (1, 2) on which the estimation method is based and the simulated model (11–13) on which the estimation accuracy is tested. First, AHP is not included in the simulated model. This difference is not essential for the evaluation of the accuracy because it is straightforward to estimate it from the voltage trace. Second, the input in the OU-AHP model is described by the current input, whereas the input in the simulated model is described by the conductance inputs.

The stimulus effect was mimicked by a rectangular window function:

(14) $$\begin{array}{l} \lambda_{E}(t)=\lambda_{E,0}+\delta \lambda_{E}\omega_{T}\left(t-t_{o}\right),\\ \lambda_{I}(t)\;=\lambda_{I,0}+\delta \lambda_{I}\omega_{T}\left(t-t_{o}\right), \end{array}$$

where λ_*E*_(*t*) and λ_*I*_(*t*) are the total synaptic input rates, λ_*E*, 0_ and λ_*I*, 0_ are the initial values, δλ_*E*_, δλ_*I*_ are the changes in the rates as a result of the stimulation, *t*_*o*_ is the stimulus onset time, ω_*T*_(*t*) is the window function: ω_*T*_(*t*) = 1 (0 < *t* < *T*), otherwise ω_*T*_(*t*) = 0, and *T* is the stimulus duration. The synaptic parameters were *N*_*E*_ = 1000, *g*_*E*_ = 1.2 nS, *V*_*E*_ = 0 mV, and τ_*E*_ = 1.0 ms for the excitatory neurons, and *N*_*I*_ = 1000, *g*_*I*_ = 3.0 nS, *V*_*I*_ = −75 mV, and τ_*I*_ = 2.0 ms for the inhibitory neurons, which were adopted from Häusser and Roth (1997) and Destexhe et al. (1998). The stimulus parameters were λ_*E*, 0_ = 1.8 kHz, λ_*I*, 0_ = 2.0 kHz, *t*_*o*_ = 0 s, and *T* = 1 s, and the remaining parameters are provided in the figure captions (**Figures 4**, **5**). Equation (11) was solved numerically using the forward Euler method with a time step of 0.01 ms. The Euler method was sufficient, because the time step was substantially shorter than all intrinsic time constants of the model.

### Experimental data and analysis

**Figure 3:**
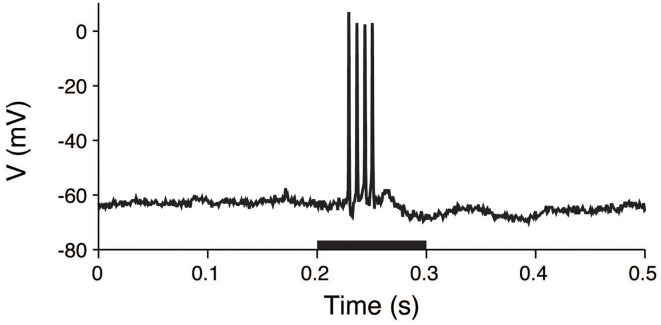
**Voltage trace of a thalamic neuron in response to an acoustic stimulus**. The membrane potential was intracellularly recorded from a thalamic neuron. The stimulus was applied during the 0.2–0.3 s period (black bar).

The procedures for animal preparations, auditory stimulation, and electrophysiological recordings have been previously reported (He, 2003). The experimental procedures were approved by the Animal Subjects Ethics Sub-Committee of The Hong Kong Polytechnic University. In brief, the membrane voltages of the guinea pig thalamic neurons were intracellularly recorded. Anesthesia was initially induced with sodium pentobarbital, and it was maintained by supplemental doses of the same anesthetic during surgical preparation and recording. Acoustic stimuli were delivered by a dynamic earphone. The subject was placed in a double-walled soundproof room, and repeated noise bursts and pure tones with a 5 ms rise/fall time were used to examine the neuronal responses. The duration of the stimuli was 0.1 s, and the interval between stimuli was at least 1.2 s. A sample voltage recording is shown in Figure 3.

We analyzed the membrane voltage of a thalamic neuron, which was recorded for 501 s with a sampling interval of 0.15 ms. The recorded voltage trace was divided into two components, the spike and the sub-threshold voltage trace. The spike component was defined as the 4.5 ms voltage traces after each spike onset. Spikes were detected if the membrane potential exceeded −30 mV. The spike onset was defined as the time when the derivative of the voltage exceeded 10 mV/ms (Azouz and Gray, 2000). The sub-threshold component was obtained by removal of the spikes from the voltage trace. The residual sub-threshold voltage trace was filtered by a 6-point moving average (Lansky et al., 2006, 2010). The effective bandwidth of the voltage trace after the moving average was between 0 and 1100 Hz. Finally, the input to the neuron was estimated from the sub-threshold voltage trace (Section Estimation of Input Signals for details).

## Results

Neuronal firing during stimulation may follow four scenarios: (1) no effect (spontaneous input prevails), (2) an excitatory effect, (3) a mixed excitatory and inhibitory effects, and (4) an inhibitory effect. We first demonstrate that the described method (Section Estimation of Input Signals) can distinguish these scenarios using the simulated data (Section Single-compartment Neuron Model with Realistic Synaptic Inputs). This method was subsequently applied to a voltage trace from an auditory thalamic neuron *in vivo*, and the effect of stimulation was examined.

### Simulated data

A realistic neuron model (Section Single-compartment Neuron Model with Realistic Synaptic Inputs) was simulated to test the method used to characterize the effect of the stimulus on the synaptic input. Prior to the analysis, we calculated the auto-correlation function from a simulated voltage trace. The auto-correlation function can be well described by the exponential function with the exception of small time lag values (Berg and Ditlevsen, 2013). To remedy the discrepancy, we sub-sampled the data: i.e., the sampling interval was set to 0.9 ms. The membrane time constant τ_*m*_ was evaluated from the auto-correlation function, τ_*m*_ = 19 ms. The EPSP, IPSP and the resting potential were determined by minimizing the error between the true synaptic input rates and their estimates, the EPSP and IPSP: *a*_*E*_ = 0.11 mV and *a*_*I*_ = 0.09 mV, which were in the range obtained by direct measurements (Magee and Cook, 2000; Song et al., 2005). The resting potential was *u*_0_ = −65.5 mV.

**Figure 4:**
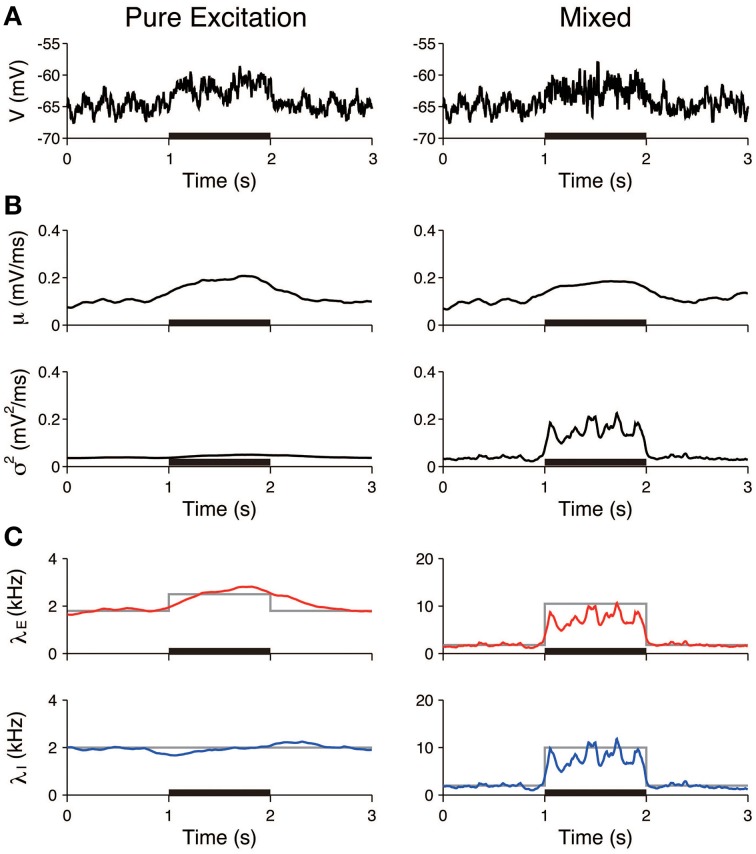
**Identification of stimulation effects from a single voltage trace: detection of pure excitation**. **(A)** Simulated voltage traces. The neuron was stimulated by pure excitation (left) or mixed excitation and inhibition (right). The stimulus was applied during the 1–2 s period (black bar). **(B)** Estimated input mean μ (top) and input variance σ^2^ (bottom). **(C)** Deduced excitatory λ_*E*_ (red) and inhibitory λ_*I*_ (blue) input rates from input mean μ and input variance σ^2^ (Equation 4). Gray lines represent the true input rates. The stimulus parameters were δλ_*E*_ = 0.7 kHz and δλ_*I*_ = 0 kHz on the left, and δλ_*E*_ = 8.7 kHz and δλ_*I*_ = 8.0 kHz on the right.

**Figure 5:**
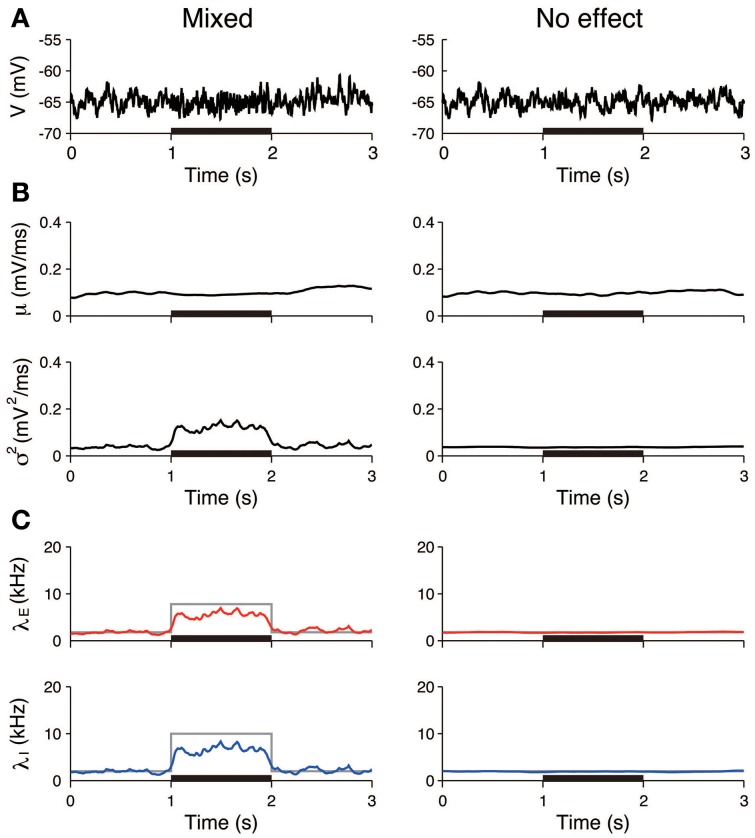
**Identification stimulation effects from a single voltage trace: detection of the mixed effects**. **(A)** Simulated voltage traces. The neuron was stimulated in accordance with the mixed effect (left) and no effect (right). The stimulus was applied during the 1–2 s period (black bar). **(B)** Estimated input mean μ (top) and input variance σ^2^ (bottom). **(C)** Deduced excitatory λ_*E*_ (red) and inhibitory λ_*I*_ (blue) input rates from input mean μ and input variance σ^2^ (Equation 4). Gray lines represent the true input rates. The stimulus parameters were δλ_*E*_ = 6.0 kHz and δλ_*I*_ = 8.0 kHz on the left, and δλ_*E*_ = 0 kHz and δλ_*I*_ = 0 kHz on the right.

The first three scenarios were considered, with the exclusion of the case of inhibition. First, we examined whether pure excitation (δλ_*E*_ > 0, δλ_*I*_ = 0) can be distinguished from mixed excitation and inhibition (δλ_*E*_ > 0, δλ_*I*_ > 0). The voltage traces generated in accordance with these two cases appeared similar (Figure 4A). The estimated input means μ were also similar in both cases and increased during the stimulation. In contrast, the estimated input variances σ^2^ differed. The variance was approximately constant in the case of the pure excitation, whereas it increased in the case of the mixed excitation and inhibition (Figure 4B). The excitatory and inhibitory input rates were calculated from the input statistics (μ, σ^2^) using Equation (4). The deduced input rates qualitatively reproduced the different effects (Figure 4C), which suggests that this method can distinguish them. Second, we examined whether the mixed effect can be distinguished from no effect. The voltage traces generated in accordance with the two cases again appeared similar (Figure 5A). The estimated input means were also similar, whereas the estimated input variances differed. The variance increased during the stimulation in the case of the mixed effect, whereas it remained constant in the case of no effect (Figure 5B). Thus, the method again detected the difference in the stimulus (Figure 5C).

Furthermore, the input rates were estimated from 50 independent simulated voltage traces. The estimated rates averaged over the stimulated period (1–2 s) were compared to those averaged over the unstimulated period (0.5–1 and 2–2.5 s). In the pure excitation scenario (Figure 4, left), the excitatory rate significantly increased (*t*-test *p* < 0.01) and the inhibitory rate decreased (*t*-test *p* < 0.01) during the stimulated period. In the mixed excitation and inhibition scenario (Figure 4, right; Figure 5, left), the excitatory and inhibitory rates increased (*t*-test *p* < 0.01) during the stimulated period. On the other hand, the excitatory and inhibitory rates did not change (*t*-test *p* > 0.05) in the case of no effect (Figure 5, right). The estimation method can detect the stimulation effects correctly except for the pure excitation scenario, i.e., the method wrongly identified the decrease in inhibition whereas the inhibition did not change. A possible reason is that the effect of conductance change is neglected in the OU-AHP model. The results suggest that the method can identify the stimulation effect on the synaptic input from a voltage trace.

### Experimental data: unstimulated period

The input to an auditory neuron during the unstimulated period was investigated. The experimental data were divided into 10 equal intervals of 15 s. Similar to the simulated data, the EPSP and IPSP were set to *a*_*E*_ = 0.11 mV and *a*_*I*_ = 0.09 mV, respectively, and the resting potential was set to *u*_0_ = −65.5 mV. The membrane time constant was determined from the auto-correlation function, τ_*m*_ = 26 ms, which is in agreement with the time constant obtained by the maximum likelihood method, τ_*m*_ = 23 ms (Lansky et al., 2006). The sampling interval was set to 0.9 ms, with the exception of the spike onsets where it was set to 4.5 ms, to eliminate the spike waveform.

**Figure 6:**
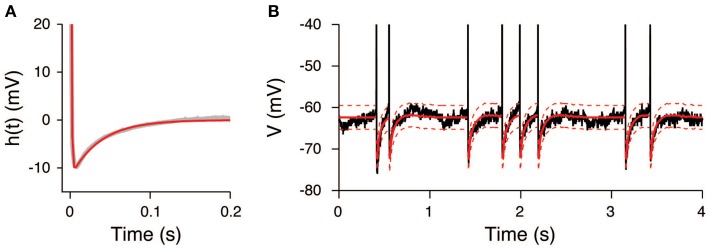
**AHP function extracted from the voltage trace during the unstimulated period**. **(A)** Extracted AHP function *h*(*t*). The AHP function (gray) was fitted by the sum of two exponential functions (red). **(B)** Comparison of the recorded voltage (black) with the mean (red) and its 95% confidence bounds (dashed red line) of the OU-AHP model.

The AHP function *h*(*t*) was calculated under the assumption that the input signals were constant during the unstimulated period. It can be well-fitted by the sum of two exponential functions (Figure 6A) with the parameters *a*_1_ = 160 mV, τ_1_ = 0.9 ms, *a*_2_ = −12 mV, and τ_2_ = 37 ms. The extracted AHP function is similar to the function obtained from a Hodgkin-Huxley type neuron model (Kobayashi and Shinomoto, 2007). The OU-AHP model (Equations 1, 2) reproduces the behavior of the recorded voltage during the unstimulated period (Figure 6B).

**Figure 7:**
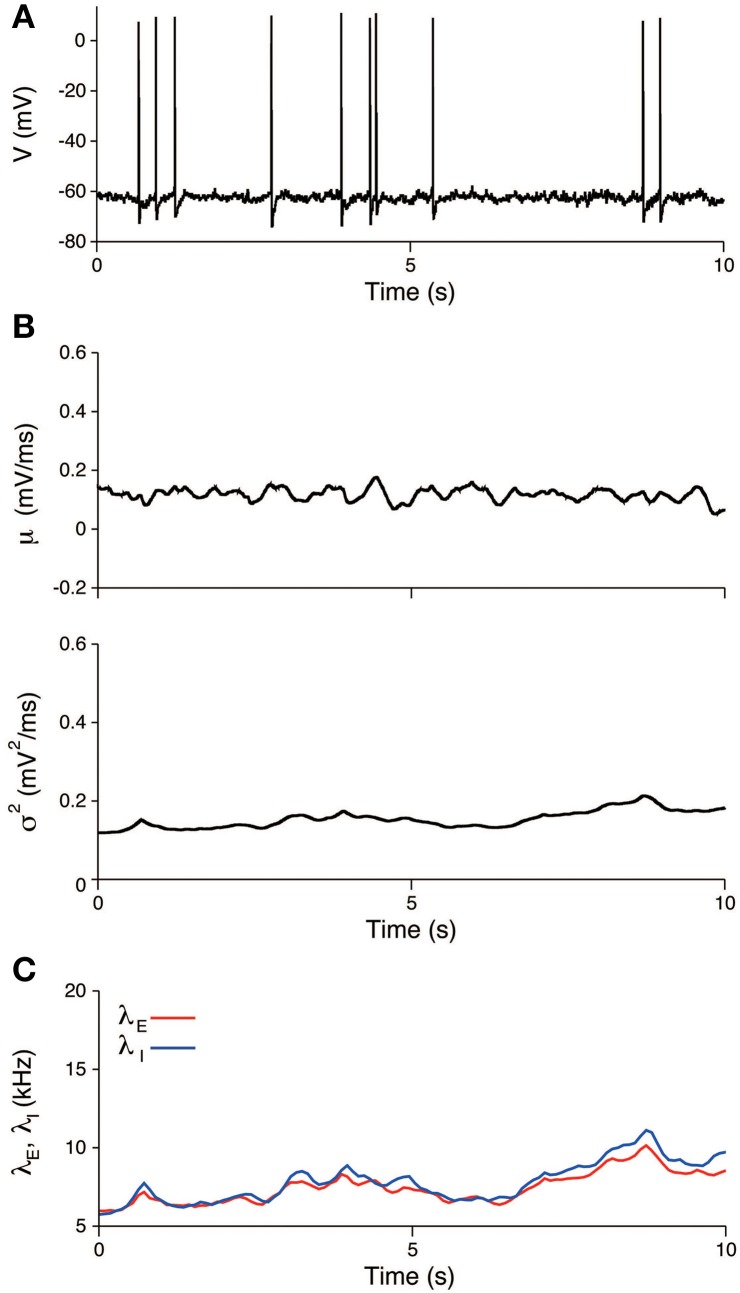
**Estimated input signals to a thalamic neuron *in vivo* during the unstimulated period**. **(A)** Voltage trace. **(B)** Estimated input mean μ (top) and input variance σ^2^ (bottom). **(C)** Deduced excitatory λ_*E*_(*t*) (red) and inhibitory λ_*I*_(*t*) (blue) input rates.

The input statistics (μ, σ^2^) were estimated from the potential *U*(*t*), which was obtained by subtracting the AHP from the voltage. An example of the estimate is shown in Figure 7B. The estimates are μ = 0.12± 0.02 mV/ms and σ^2^ = 0.16± 0.03 mV^2^/ms. All quantities represent the mean ± SD, unless stated otherwise. The input variance σ^2^ was close to the estimate of the maximum likelihood method, 0.18 mV^2^/ms (Lansky et al., 2006). In contrast, the input mean μ differed from the estimate of the maximum likelihood method, 0.46 mV/ms. This difference is because the resting potential *u*_0_ was fixed at —65.5 mV in our study, whereas it was considered to be the minimum voltage within each inter-spike interval in the cited paper. It is possible to obtain the more concordant values by decreasing the resting potential in our model. For example, if the resting potential was set to −74 mV, the input mean was 0.45 ± 0.02 mV/ms. The estimate of the inhibitory synaptic input rate was slightly higher than the excitatory rate (Figure 7C). The average input rates were λ_*E*_ = 7.9± 1.3 kHz and λ_*I*_ = 8.3± 1.6. The input rates were strongly correlated (*r* = 0.99, data not shown), which indicates that the excitation and inhibition were balanced during the unstimulated period.

### Experimental data: stimulated period

**Figure 8:**
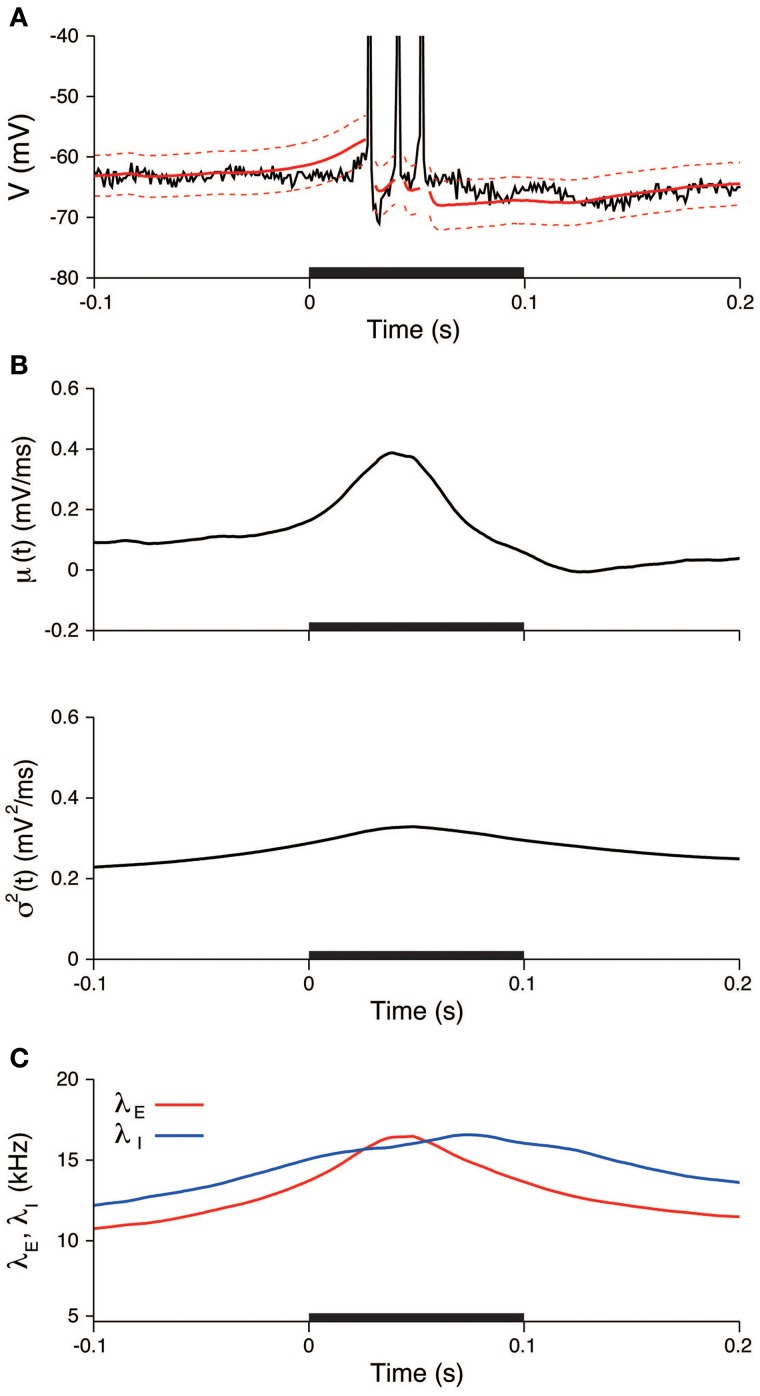
**An example of the estimated input signals to a thalamic neuron *in vivo* during a stimulation**. **(A)** Voltage trace. The recorded voltage (black) was compared with the mean (red) and its 95% confidence bounds (dashed red line) of the OU-AHP model. **(B)** Estimated input mean μ (top) and input variance σ^2^ (bottom). **(C)** Deduced excitatory λ_*E*_(*t*) (red) and inhibitory λ_*I*_(*t*) (blue) input rates. The stimulus duration (0-0.1 s) is indicated by the black bar.

In the same way as in the previous section, the input was also estimated during the stimulation. The EPSP and IPSP, the membrane time constant, the resting potential, and the sampling interval were taken as before. The recorded voltage was compared with the OU-AHP model (Equations 1, 2). The 95% confidence bounds for the model covers the recorded voltage almost completely (Figure 8A). In the trial, both the input mean μ and variance σ^2^ increased during the stimulation and then returned to their spontaneous values (Figure 8B). Consequently, the excitatory and inhibitory input rates followed the same time dependency (Figure 8C).

**Figure 9:**
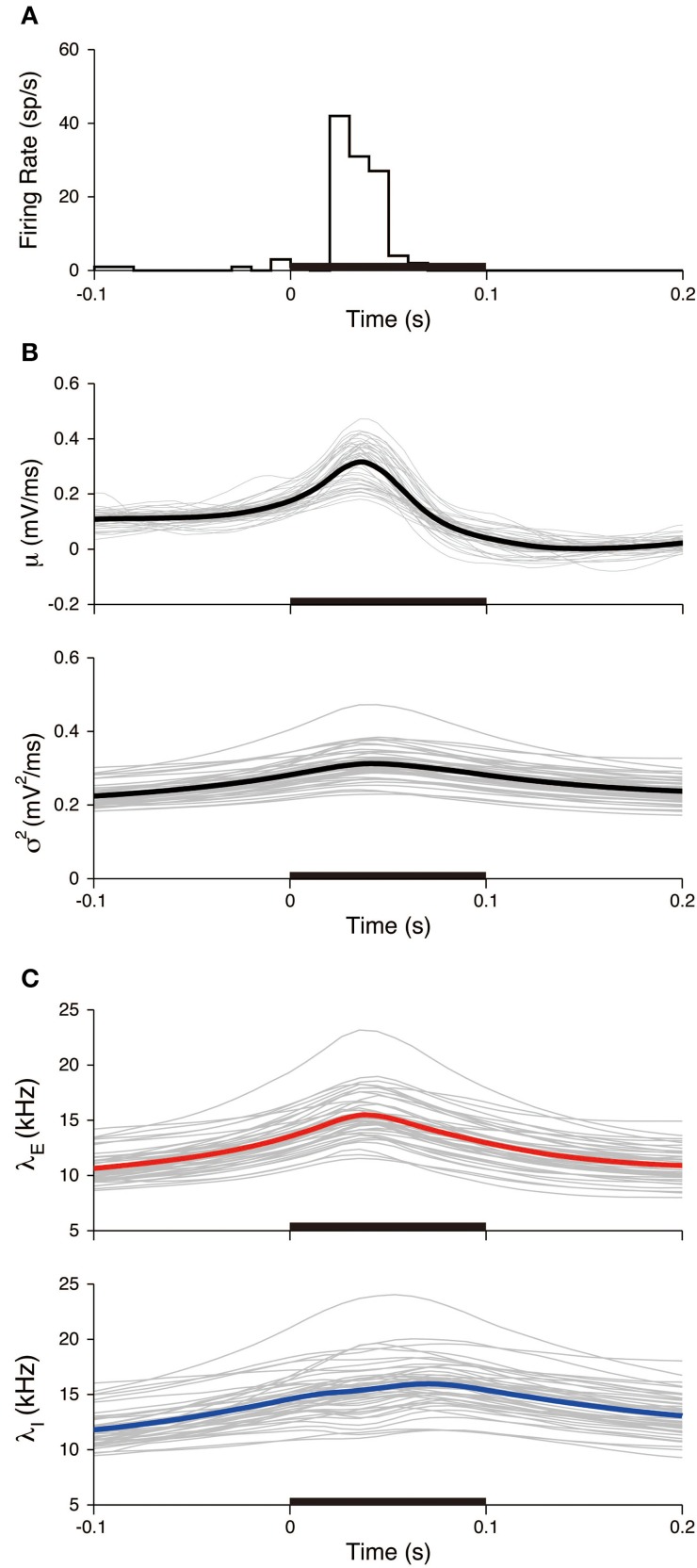
**Stimulus-triggered average of input signals. (A)** PSTH of the neuron. The bin width was 10 ms. **(B)** Estimated input mean μ (top) and input variance σ^2^ (bottom). Gray lines represent individual estimates from each trial, black lines represent their averages over the trials. **(C)** Deduced excitatory λ_*E*_(*t*) (top) and inhibitory λ_*I*_(*t*) (bottom) input rates. Gray lines represent the input rates deduced from each trial, and blue (red) lines represent the averages of the excitatory (inhibitory) rates over the trials. The stimulus duration (0-0.1 s) is indicated by the black bar.

All the experimental trials were used to evaluate the variability of the neuronal responses within the stimulated period. The peristimulus time histogram (PSTH) and the stimulus-triggered average of the input signals were calculated (Figure 9). The PSTH is defined in a standard way as a histogram of spike times from stimulus onset. The stimulus-triggered average of a signal *s*(*t*) is defined as an average over the trials:

$$s_{\text{STA}}(t)=:\frac{1}{K}\sum_{j\;=\;1}^{K}s\left(t+t_{j}\right),$$

where *K* = 45 is the number of trials, *t* is the relative time from the stimulus onset, and *t*_*j*_ is the j-th stimulus onset time. The PSTH has a sharp peak (Figure 9A), which indicates that spikes are reliably induced after stimulus onset. Both the averaged mean μ_STA_(*t*) and variance σ^2^_STA_(*t*) increased during the stimulation (Figure 9B). The increase in the variance was modest compared with the mean. The input mean and variance reached a maximum at 36 and 41 ms, respectively, after the stimulus onset. The excitatory synaptic input rate increased faster than the inhibitory rate. The excitation and inhibition achieved their maximal rates at 38 and 70 ms, respectively, after the stimulus onset (Figure 9C). The changes in the rates because of the stimulation were δλ_*E*_ = 7.6 kHz and δλ_*I*_ = 7.7 kHz. Both input rates roughly doubled in their activities.

**Figure 10:**
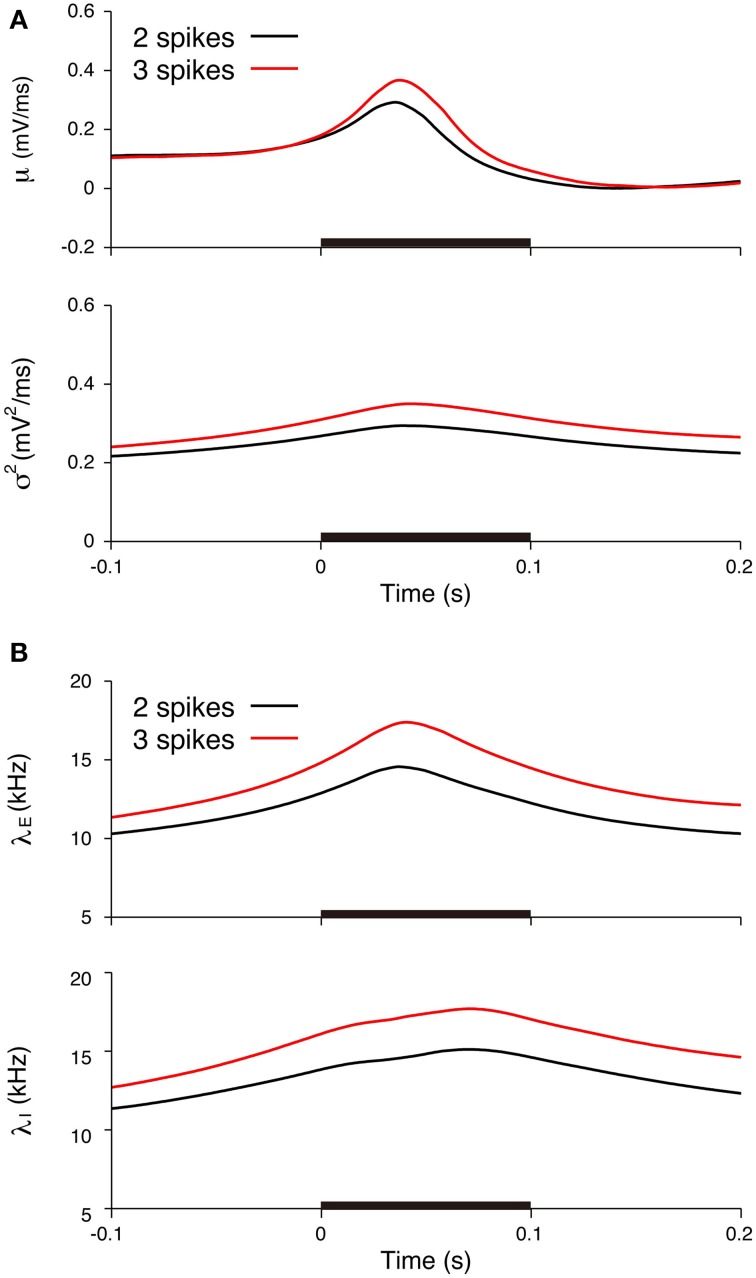
**Comparisons of the input signals between the two-spike and three-spike trials**. **(A)** Estimated input mean μ (top) and input variance σ^2^ (bottom). **(B)** Deduced excitatory λ_*E*_(*t*) (top) and inhibitory λ_*I*_(*t*) (bottom) input rates. Black lines represent the averages for the two-spike trials, and red lines represent the averages for the three-spike trials. The stimulus duration (0-0.1 s) is indicated by the black bar.

We subsequently examined the relationship between the deduced inputs and the spike response. The spike response of a neuron was characterized by the number of spikes during stimulation. The neuron generated two spikes (29 trials) or three spikes (14 trials) in most cases. The input mean and variance averaged over the trials with three spikes (three-spike trials) increased more than the values averaged over the two-spike trials (Figure 10A). The input variance averaged over the three-spike trials was higher than the two-spike trials even before the stimulation. The reason for the difference before the stimulation may be because of the smoothing effect in the estimation method. The maximum input rates averaged over the three-spike trials were also higher than the rates averaged over the two-spike trials, λ_*E*_ = 14.6 kHz and λ_*I*_ = 15.1 kHz for the two-spike trials and λ_*E*_ = 17.4 kHz and λ_*I*_ = 17.7 kHz for the three-spike trials (Figure 10B).

## Discussion

We have developed a method to characterize the stimulus effect on the synaptic input from a single voltage trace (Figure 2). First, the method was tested using simulated data generated by a single-compartment neuron model with conductance inputs (Figures 4, 5). Application to a recording from a thalamic auditory neuron *in vivo* suggested that excitatory and inhibitory inputs do not vary markedly during the unstimulated period (Figure 7) but increase during stimulation (Figures 8, 9). Thus, the findings indicate that the excitatory synaptic inputs are balanced with the inhibition and that the excitation is faster than the inhibition.

There are three essential assumptions that underlie the applied method. First, it is based on the OU-AHP model (Section OU Model with AHP and Characterization of the Stimulus Effect), and it is assumed that the synaptic input is described by the current input and the activities of presynaptic neurons are independent. The conductance input could affect the voltage fluctuations in a different way. For example, an increase in inhibition always increases the voltage fluctuations in the OU-AHP model, whereas it can decrease the voltage fluctuations in the conductance input model and in the experiment (Monier et al., 2003). This factor can explain the reason why the estimated inhibitory rate slightly decreased during the stimulation in the pure excitation scenario (Figure 4), and the input rates were underestimated in the mixed excitation and inhibition scenario (Figures 4, 5). However, the current input model is a good approximation of the conductance model if the voltage fluctuations are not large. This argument suggests that the estimation method can work for the voltage traces with small fluctuations, including the simulated and experimental data in this study. Second, the input statistics are assumed to vary smoothly. Although hyperparameters that control the variability of the input statistics were tuned from the data using the EM algorithm (Supplementary Material, Appendix A), the estimation method may not capture a rapid change, including a subtle difference between the onsets of excitation and inhibition (Wehr and Zador, 2003). Nevertheless, our method has suggested that the excitatory and inhibitory inputs to an auditory neuron are balanced and the increase in excitation is faster than that in inhibition (Figure 9). The results are consistent with the findings of the conductance estimation of auditory neurons in anesthetized rats (Wehr and Zador, 2003). Third, the amplitudes of EPSP and IPSP were determined from the simulated data, and they were fixed for the analysis of the experimental data. The estimates of EPSP and IPSP (*a*_*E*_ = 0.11 mV and *a*_*I*_ = 0.09 mV) were consistent with the results of other experimental studies (Magee and Cook, 2000; Song et al., 2005); however, there is no way to determine the validity.

It should be noted that the neuron model used for simulation may not capture important properties of a neuron *in vivo*. First, it does not have the active ionic currents that contribute to spike generation. Second, it neglects the effects of the spatial properties of a neuron, i.e., the dendrite and the axon. Third, the activities of pre-synaptic neurons obey the independent Poisson process. The estimation method could be further verified via comparisons with the voltage-clamp data or applying it to experimental data with pharmacological manipulations (Berg and Ditlevsen, 2013). However, this validation is beyond the scope of this study.

Our method can deduce the synaptic input rates (λ_*E*_, λ_*I*_), which are equal to the sum of the firing rates of all presynaptic neurons. The estimates were λ_*E*_ = 8 kHz and λ_*I*_ = 8 kHz during the unstimulated period, which then doubled during the stimulation. The number of presynaptic neurons can be estimated to be 5000 for excitatory neurons and 1000 for inhibitory neurons (Braitenberg and Schuz, 1998). This estimate indicates that the average spontaneous firing rates were 1.6 and 8 Hz for individual excitatory and inhibitory neurons, respectively, and the values are doubled during the stimulation. We also demonstrated that the estimated input rates in the three-spike trials were higher than in the two-spike trials (Figure 10). The findings suggest that the spike response is controlled by the intensity of the excitation and inhibition.

It has been reported that synaptic inputs with balanced excitation and inhibition are common in the visual cortex (Borg-Graham et al., 1998), auditory cortex (Wehr and Zador, 2003), cerebral cortex (Rudolph et al., 2007), and spinal cord (Berg et al., 2007). Our results indicate that the synaptic input to a thalamic auditory neuron is also balanced, which is consistent with a previous study based on voltage-clamp recordings *in vivo* (Wehr and Zador, 2003). Balanced inputs are considered functionally important because they can improve the efficacy of stimulus coding. First, the balanced inputs can facilitate spike rate coding via the modulation of the stimulus response curve (*f–I* curve) (Lansky and Sacerdote, 2001; Kobayashi, 2009; Sutherland et al., 2009). The simultaneous increase in excitation and inhibition can enlarge the coding range of the neuron. Second, the balanced inputs can facilitate spike time coding via improvements in the reliability of spike generation (Wehr and Zador, 2003). Indeed, the auditory neuron reliably generates spikes after stimulus onset (Figure 9A). It is also suggested that the balanced inputs can improve the decoding performance of the stimulus from a spike train by clamping the firing irregularity of a neuron (Miura et al., 2007).

### Conflict of interest statement

The authors declare that the research was conducted in the absence of any commercial or financial relationships that could be construed as a potential conflict of interest.

## References

[B1] Abbott L. F. Kepler T. (1990). Model neurons: from Hodgkin-Huxley to Hopfield, in Statistical Mechanics of Neural Networks, ed Garrido L. (Berlin: Springer-Verlag), 5–18.

[B2] Azouz R. Gray C. M. (2000). Dynamic spike threshold reveals a mechanism for synap- tic coincidence detection in cortical neurons *in vivo*. Proc. Natl. Acad. Sci. U.S.A. 97, 8110–8115. 10.1073/pnas.130200797 10859358 PMC16678

[B3] Bedard C. Behuret S. Deleuze C. Bal T. Destexhe A. (2012). Oversampling method to extract excitatory and inhibitory conductances from single-trial membrane potential recordings. J. Neurosci. Method 210, 3–14. 10.1016/j.jneumeth.2011.09.010 21968037

[B4] Berg R. W. Alaburda A. Hounsgaard J. (2007). Balanced inhibition and excitation drive spike activity in spinal half-centers. Science 315, 390–393. 10.1126/science.1134960 17234950

[B5] Berg R. W. Ditlevsen S. (2013). Synaptic inhibition and excitation estimated via the time constant of membrane potential fluctuations. J. Neurophysiol. 110, 1021–1034. 10.1152/jn.00006.2013 23636725

[B6] Borg-Graham L. J. Monier C. Fregnac Y. (1998). Visual input evokes transient and strong shunting inhibition in visual cortical neurons. Nature 393, 369–373. 10.1038/30735 9620800

[B7] Braitenberg V. Schuz A. (1998). Cortex: Statistics and Geometry of Neuronal Connectivity. Berlin: Springer.

[B8] Destexhe A. Mainen Z. Sejnowski T. J. (1998). Kinetic models of synaptic transmission, in Methods in Neuronal Modeling, eds Koch C. Segev I. (Cambridge, MA: MIT Press), 1–26.

[B9] Destexhe A. (1997). Conductance-based integrate-and-fire models. Neural Comput. 9, 503–514. 10.1162/neco.1997.9.3.503 9097470

[B10] Ditlevsen S. Lansky P. (2005). Estimation of the input parameters in the Ornstein—Uhlenbeck neuronal model. Phys. Rev. E 71:011907. 10.1103/PhysRevE.71.011907 15697630

[B11] Gerstner W. Kistler W. M. (2002). Spiking Neuron Models: Single Neurons, Populations, Plasticity. Cambridge: Cambridge University Press.

[B12] Häusser M. Roth A. (1997). Estimating the time course of the excitatory synaptic conductance in neocortical pyramidal cells using a novel voltage jump method. J. Neurosci. 17, 7606–7625. 9315883 10.1523/JNEUROSCI.17-20-07606.1997 PMC6793890

[B13] He J. (2003). Slow oscillation in non-lemniscal auditory thalamus. J. Neurosci. 23, 8281–8290. 12967990 10.1523/JNEUROSCI.23-23-08281.2003 PMC6740700

[B14] Jolivet R. Lewis T. J. Gerstner W. (2004). Generalized integrate-and-fire models of neuronal activity approximate spike trains of a detailed model to a high degree of accuracy. J. Neurophysiol. 92, 959–976. 10.1152/jn.00190.2004 15277599

[B15] Kim H. Shinomoto S. (2012). Estimating nonstationary input signals from a single neuronal spike train. Phys. Rev. E 86:051903. 10.1103/PhysRevE.86.051903 23214810

[B16] Kitagawa G. Gersch W. (1996). Smoothness Priors Analysis of Time Series. Lecture Notes in Statistics, *Vol. 116* New York, NY: Springer-Verlag.

[B17] Kobayashi R. Shinomoto S. (2007). State space method for predicting the spike times of a neuron. Phys. Rev. E 75:011925. 10.1103/PhysRevE.75.011925 17358202

[B18] Kobayashi R. Shinomoto S. Lansky P. (2011a). Estimation of time-dependent input from neuronal membrane potential. Neural Comput. 23, 3070–3093. 10.1162/NECO_a_00205 21919789

[B19] Kobayashi R. Tsubo Y. Lansky P. Shinomoto S. (2011b). Estimating time-varying input signals and ion channel states from a single voltage trace of a neuron. Adv. Neural Inform. Process. Syst. 24, 217–225.

[B20] Kobayashi R. (2009). The influence of firing mechanisms on gain modulation. J. Stat. Mech. 2009:P01017 10.1088/1742-5468/2009/01/P01017

[B21] Koyama S. Shinomoto S. (2005). Empirical Bayes interpretations of random point events. J. Physics A 38, 531–537. 10.1088/0305-4470/38/29/L0417321039

[B22] Lankarany M. Zhu W. P. Swamy M. N. S. Toyoizumi T. (2013). Inferring trial-to-trial excitatory and inhibitory synaptic inputs from membrane potential using Gaussian mixture Kalman filtering. Front. Comput. Neurosci. 7:109 10.3389/fncom.2013.00109 PMC3759749 24027523

[B23] Lansky P. Ditlevsen S. (2008). A review of the methods for signal estimation in stochastic diffusion leaky integrate-and-fire neuronal models. Biol. Cybern. 99, 253–262. 10.1007/s00422-008-0237-x 18496710

[B24] Lansky P. Musila M. Smith C. E. (1992). Effects of afterhyperpolarization on neuronal firing. Biosystems 27, 25–38. 10.1016/0303-2647(92)90044-Y 1391689

[B25] Lansky P. Sacerdote L. (2001). The Ornstein-Uhlenbeck neuronal model with signal-dependent noise. Phys. Lett. A 285, 132–140. 10.1016/S0375-9601(01)00340-1 12459301

[B26] Lansky P. Sanda P. He J. (2006). The parameters of the stochastic leaky integrate-and-fire neuronal model. J. Comput. Neurosci. 21, 211–223. 10.1007/s10827-006-8527-6 16871351

[B27] Lansky P. Sanda P. He J. (2010). Effect of stimulation on the input parameters of stochastic leaky integrate-and-fire neuronal model. J. Physiol. 104, 160–166. 10.1016/j.jphysparis.2009.11.019 19944155

[B28] Lansky P. (1983). Inference for the diffusion models of neuronal activity. Math. Biosci. 67, 247–260. 10.1016/0025-5564(83)90103-719188601

[B29] Magee J. C. Cook E. P. (2000). Somatic EPSP amplitude is independent of synapse location in hippocampal pyramidal neurons. Nature Neurosci. 3, 895–903. 10.1038/78800 10966620

[B30] Miura K. Tsubo Y. Okada M. Fukai T. (2007). Balanced excitatory and inhibitory inputs to cortical neurons decouple firing irregularity from rate modulations. J. Neurosci. 27, 13802–13812. 10.1523/JNEUROSCI.2452-07.2007 18077692 PMC6673628

[B31] Monier C. Chavane F. Baudot P. Graham L. J. Fregnac Y. (2003). Orientation and direction selectivity of synaptic inputs in visual cortical neurons: a diversity of combinations produces spike tuning. Neuron 37, 663–680. 10.1016/S0896-6273(03)00064-3 12597863

[B32] Monier C. Fournier J. Fregnac Y. (2008). *In vitro* and *in vivo* measures of evoked excitatory and inhibitory conductance dynamics in sensory cortices. J. Neurosci. Methods 169, 323–365. 10.1016/j.jneumeth.2007.11.008 18215425

[B33] Paninski L. Vidne M. DePasquale B. Ferreira D. G. (2012). Inferring synaptic inputs given a noisy voltage trace via sequential Monte Carlo methods. J. Comput. Neurosci. 33, 1–19. 10.1007/s10827-011-0371-7 22089473

[B34] Rudolph M. Piwkowska Z. Badoual M. Bal T. Destexhe A. (2004). A method to estimate synaptic conductances from membrane potential fluctuations. J. Neurophysiol. 91, 2884–2896. 10.1152/jn.01223.2003 15136605

[B35] Rudolph M. Pospischil M. Timofeev I. Destexhe A. (2007). Inhibition determines membrane potential dynamics and controls action potential generation in awake and sleeping cat cortex. J. Neurosci. 27, 5280–5290. 10.1523/JNEUROSCI.4652-06.2007 17507551 PMC6672346

[B36] Shadlen M. N. Newsome W. T. (1998). The variable discharge of cortical neurons: implications for connectivity, computation, and information coding. J. Neurosci. 18, 3870–3896. 9570816 10.1523/JNEUROSCI.18-10-03870.1998 PMC6793166

[B37] Shinomoto S. Sakai Y. Funahashi S. (1999). The Ornstein-Uhlenbeck process does not reproduce spiking statistics of neurons in prefrontal cortex. Neural Comput. 11, 935–951. 10.1162/089976699300016511 10226190

[B38] Smith A. C. Scalon J. D. Wirth S. Yanike M. Suzuki W. A. Brown E. N. (2010). State-space algorithms for estimating spike rate functions. Comput. Intell. Neurosci. 2010:426539 10.1155/2010/426539 19911062 PMC2774470

[B39] Softky W. R. Koch C. (1993). The highly irregular firing of cortical cells is inconsistent with temporal integration of random EPSPs. J. Neurosci. 13, 334–350. 8423479 10.1523/JNEUROSCI.13-01-00334.1993 PMC6576320

[B40] Song S. Sjöström P. J. Reigl M. Nelson S. Chklovskii D. B. (2005). Highly nonrandom features of synaptic connectivity in local cortical circuits. PLoS Biol. 3:e68. 10.1371/journal.pbio.0030068 15737062 PMC1054880

[B41] Sutherland C. Doiron B. Longtin A. (2009). Feedback-induced gain control in stochastic spiking networks. Biol. Cybern. 100, 475–489. 10.1007/s00422-009-0298-5 19259695

[B42] Tuckwell H. C. (1988). Introduction to Theoretical Neurobiology: Nonlinear and Stochastic Theories, Vol. 2. Cambridge: Cambridge University Press.

[B43] Wehr M. Zador A. M. (2003). Balanced inhibition underlies tuning and sharpens spike timing in auditory cortex. Nature 426, 442–446. 10.1038/nature02116 14647382

