# Supplementary material for: Estimation of the synaptic input firing rates and characterization of the stimulation effects in an auditory neuron
Source: Front Comput Neurosci. 2015 May 18;9:59. doi: 10.3389/fncom.2015.00059 (PMC4435043; doi:10.3389/fncom.2015.00059)
Supplement: Supplementary file 1 [file DataSheet1.DOCX]

***Supplementary Material***

**Estimation of the synaptic input firing rates and characterization of the stimulation effects in an auditory neuron**

**Ryota Kobayashi*, Jufang He, Petr Lansky**

*** Correspondence:** Ryota Kobayashi, Principles of Informatics Research Division, National Institute of Informatics, 2-1-2 Hitotsubashi, Chiyoda-ku, Tokyo, 101-8430, Japan.

[r-koba@nii.ac.jp](mailto:r-koba@nii.ac.jp)

**Appendix A: Tuning hyperparameters**

The hyperparameters $\vec{\boldsymbol{\theta}}\boldsymbol{=(}\boldsymbol{\gamma}_{\boldsymbol{M}}\boldsymbol{,}\boldsymbol{\gamma}_{\boldsymbol{S}}\boldsymbol{)}$ were tuned using EM algorithm, and the procedure is subsequently described. The EM algorithm is commonly used to estimate parameters in a model that has both observable and latent variables. In our model (9), the observable variables are the changes in the voltage $\boldsymbol{Z}_{\boldsymbol{1}}\boldsymbol{,}\boldsymbol{Z}_{\boldsymbol{2}}\boldsymbol{,\cdots,}\boldsymbol{Z}_{\boldsymbol{N-1}}$, and the latent variables are the input signals ${\vec{\boldsymbol{X}}}_{\boldsymbol{1}}\boldsymbol{,}{\vec{\boldsymbol{X}}}_{\boldsymbol{2}}\boldsymbol{,\cdots,}{\vec{\boldsymbol{X}}}_{\boldsymbol{N-1}}$. The hyperparameters $\vec{\boldsymbol{\theta}}$ are determined via maximization of the expected value of the log likelihood function ($\boldsymbol{Q}$ function),

$${\vec{\boldsymbol{\theta}}}_{\boldsymbol{k+1}}\boldsymbol{=}\underset{\vec{\boldsymbol{\theta}}}{\mathbf{argmax}} \boldsymbol{Q(}\vec{\boldsymbol{\theta}}\boldsymbol{|}{\vec{\boldsymbol{\theta}}}_{\boldsymbol{k}}\boldsymbol{)}\boldsymbol{,}(15)$$

where $\boldsymbol{Q}\left( \vec{\boldsymbol{\theta}} | {\vec{\boldsymbol{\theta}}}_{\boldsymbol{k}} \right)\boldsymbol{:=E}\left[ \mathbf{log}\left( \boldsymbol{P}\left[ \boldsymbol{Z}_{\boldsymbol{1}}\boldsymbol{,\cdots,}\boldsymbol{Z}_{\boldsymbol{N-1}}\boldsymbol{;}{\vec{\boldsymbol{X}}}_{\boldsymbol{1}}\boldsymbol{,\cdots,}{\vec{\boldsymbol{X}}}_{\boldsymbol{N-1}}\boldsymbol{|}\vec{\boldsymbol{\theta}} \right] \right)\boldsymbol{|}\boldsymbol{Z}_{\boldsymbol{1}}\boldsymbol{,\cdots,}\boldsymbol{Z}_{\boldsymbol{N-1}}\boldsymbol{;}{\vec{\boldsymbol{\theta}}}_{\boldsymbol{k}} \right]$ and ${\vec{\boldsymbol{\theta}}}_{\boldsymbol{k}}$ is the k-th iterated estimate of $\vec{\boldsymbol{\theta}}$. The $\boldsymbol{Q}$ function can be written as follows:

$$\boldsymbol{Q}\left( \vec{\boldsymbol{\theta}} | {\vec{\boldsymbol{\theta}}}_{\boldsymbol{k}} \right)\boldsymbol{:=}\sum_{\boldsymbol{j=1}}^{\boldsymbol{N-1}} \boldsymbol{E}\left[ \mathbf{log}\left( \boldsymbol{P}\left[ \boldsymbol{Z}_{\boldsymbol{j}}\boldsymbol{|}{\vec{\boldsymbol{X}}}_{\boldsymbol{j}} \right] \right)\boldsymbol{|}\boldsymbol{Z}_{\boldsymbol{1}}\boldsymbol{,\cdots,}\boldsymbol{Z}_{\boldsymbol{N-1}}\boldsymbol{;}{\vec{\boldsymbol{\theta}}}_{\boldsymbol{k}} \right]$$

$$\boldsymbol{+}\sum_{\boldsymbol{j=1}}^{\boldsymbol{N-2}} \boldsymbol{E}\left[ \mathbf{log}\left( \boldsymbol{P}\left[ {\vec{\boldsymbol{X}}}_{\boldsymbol{j+1}}\boldsymbol{|}{\vec{\boldsymbol{X}}}_{\boldsymbol{j}} \right] \right)\boldsymbol{|}\boldsymbol{Z}_{\boldsymbol{1}}\boldsymbol{,\cdots,}\boldsymbol{Z}_{\boldsymbol{N-1}}\boldsymbol{;}{\vec{\boldsymbol{\theta}}}_{\boldsymbol{k}} \right]\boldsymbol{+const.}(16)$$

${\vec{\boldsymbol{\theta}}}_{\boldsymbol{k+1}}$ is is determined via maximization of the $\boldsymbol{Q}$ function with respect to the hyperparameters:

$$\frac{\boldsymbol{\partial Q}}{\boldsymbol{\partial}\boldsymbol{\gamma}_{\boldsymbol{M}}^{\boldsymbol{2}}}\boldsymbol{=0,}\frac{\boldsymbol{\partial Q}}{\boldsymbol{\partial}\boldsymbol{\gamma}_{\boldsymbol{S}}^{\boldsymbol{2}}}\boldsymbol{=0.}$$

The iterative formulas are given as follows:

$$\boldsymbol{\gamma}_{\boldsymbol{M,k+1}}^{\boldsymbol{2}}\boldsymbol{=}\frac{\boldsymbol{1}}{\boldsymbol{N-2}}\sum_{\boldsymbol{j=1}}^{\boldsymbol{N-2}} \boldsymbol{E}\left[ \left( \boldsymbol{M}_{\boldsymbol{j+1}}\boldsymbol{-}\boldsymbol{M}_{\boldsymbol{j}} \right)^{\boldsymbol{2}}\boldsymbol{|}\boldsymbol{Z}_{\boldsymbol{1}}\boldsymbol{,\cdots,}\boldsymbol{Z}_{\boldsymbol{N-1}}\boldsymbol{;}{\vec{\boldsymbol{\theta}}}_{\boldsymbol{k}} \right]\boldsymbol{/}\boldsymbol{\Delta}_{\boldsymbol{j}}\boldsymbol{,}(17)$$

$$\boldsymbol{\gamma}_{\boldsymbol{S,k+1}}^{\boldsymbol{2}}\boldsymbol{=}\frac{\boldsymbol{1}}{\boldsymbol{N-2}}\sum_{\boldsymbol{j=1}}^{\boldsymbol{N-2}} \boldsymbol{E}\left[ \left( \boldsymbol{S}_{\boldsymbol{j+1}}\boldsymbol{-}\boldsymbol{S}_{\boldsymbol{j}} \right)^{\boldsymbol{2}}\boldsymbol{|}\boldsymbol{Z}_{\boldsymbol{1}}\boldsymbol{,\cdots,}\boldsymbol{Z}_{\boldsymbol{N-1}}\boldsymbol{;}{\vec{\boldsymbol{\theta}}}_{\boldsymbol{k}} \right]\boldsymbol{/}\boldsymbol{\Delta}_{\boldsymbol{j}}\boldsymbol{,}(18)$$

where $\boldsymbol{\gamma}_{\boldsymbol{M,k}}^{\boldsymbol{2}}$ and $\boldsymbol{\gamma}_{\boldsymbol{S,k}}^{\boldsymbol{2}}$ are the k-th iterated estimates of $\boldsymbol{\gamma}_{\boldsymbol{M}}^{\boldsymbol{2}}$ and $\boldsymbol{\gamma}_{\boldsymbol{S}}^{\boldsymbol{2}}$, respectively. As the EM algorithm increases the marginal likelihood at each iteration, the estimate converges to a local maximum. The conditional expectations in equations (17) and (18) are calculated using Kalman filtering and smoothing algorithm (**Kitagawa and Gersch,** 1996; **Koyama and Shinomoto,** 2005; **Smith et al.,** 2010; **Kobayashi et al.,** 2011a): see Appendix B.

**Appendix B:**  **Kalman Filtering and Smoothing Algorithm**

Here we summarize how to calculate the conditional expectations in equations (10), (17) and (18). For notational simplicity, $\vec{x}_{j|n}$, $\Sigma_{j|n}$, and $\Sigma_{i, j|n}$ are defined as follows:

$${\vec{\boldsymbol{x}}}_{\boldsymbol{j|n}}\boldsymbol{:=E}\left[ {\vec{\boldsymbol{X}}}_{\boldsymbol{j}}\boldsymbol{|}\boldsymbol{Z}_{\boldsymbol{1:n}} \right]\boldsymbol{,} (19)$$

$$\boldsymbol{\Sigma}_{\boldsymbol{j|n}}\boldsymbol{:=E}\left[ \left( {\vec{\boldsymbol{X}}}_{\boldsymbol{j}}\boldsymbol{-}{\vec{\boldsymbol{x}}}_{\boldsymbol{j|n}} \right)\left( {\vec{\boldsymbol{X}}}_{\boldsymbol{j}}\boldsymbol{-}{\vec{\boldsymbol{x}}}_{\boldsymbol{j|n}} \right)^{\boldsymbol{T}}\boldsymbol{|}\boldsymbol{Z}_{\boldsymbol{1:n}} \right]\boldsymbol{,} (20)$$

$$\boldsymbol{\Sigma}_{\boldsymbol{i,j|n}}\boldsymbol{:=E}\left[ \left( {\vec{\boldsymbol{X}}}_{\boldsymbol{i}}\boldsymbol{-}{\vec{\boldsymbol{x}}}_{\boldsymbol{i|n}} \right)\left( {\vec{\boldsymbol{X}}}_{\boldsymbol{j}}\boldsymbol{-}{\vec{\boldsymbol{x}}}_{\boldsymbol{j|n}} \right)^{\boldsymbol{T}}\boldsymbol{|}\boldsymbol{Z}_{\boldsymbol{1:n}} \right]\boldsymbol{,} (21)$$

where $\boldsymbol{Z}_{\boldsymbol{1:n}}\boldsymbol{=}\boldsymbol{Z}_{\boldsymbol{1}}\boldsymbol{,}\boldsymbol{Z}_{\boldsymbol{2}}\boldsymbol{,\cdots,}\boldsymbol{Z}_{\boldsymbol{n}}$. First, the Bayesian estimator at the final time step, $\vec{x}_{N-1|N-1}$, was calculated using prediction and filtering algorithms. Second, the Bayesian estimators at any time step, $\vec{x}_{j|N-1} \left( j=1,\cdots,N-1 \right)$, were obtained using a smoothing algorithm. Finally, the iterative formulas of the EM algorithm (17, 18) are computed using a covariance algorithm. The detailed procedure is subsequently described.

**Prediction Algorithm**

The mean and covariance of the predictive distribution $\vec{x}_{j+1|j}$, $\Sigma_{j+1|j}$ are calculated from those of the filtered distribution $\vec{x}_{j|j}$, $\Sigma_{j|j}$:

$${\vec{\boldsymbol{x}}}_{\boldsymbol{j+1|j}}\boldsymbol{=}{\vec{\boldsymbol{x}}}_{\boldsymbol{j|j}}\boldsymbol{,} (22)$$

$$\boldsymbol{\Sigma}_{\boldsymbol{j+1|j}}\boldsymbol{=}\boldsymbol{\Sigma}_{\boldsymbol{j|j}}\boldsymbol{+G,} (23)$$

where $\boldsymbol{G}$ is the covariance matrix of ${\vec{\boldsymbol{\xi}}}_{\boldsymbol{j}}$ in equation (9).

**Filtering Algorithm**

Using the Bayes theorem, the filtered distribution $\boldsymbol{P}\left[ {\vec{\boldsymbol{X}}}_{\boldsymbol{j}}\boldsymbol{|}\boldsymbol{Z}_{\boldsymbol{1:j}} \right]$ is written as follows:

$$\boldsymbol{P}\left[ {\vec{\boldsymbol{X}}}_{\boldsymbol{j}}\boldsymbol{|}\boldsymbol{Z}_{\boldsymbol{1:j}} \right]\boldsymbol{=}\frac{\boldsymbol{P}\left[ \boldsymbol{Z}_{\boldsymbol{j}}\boldsymbol{|}{{\vec{\boldsymbol{X}}}_{\boldsymbol{j}}\boldsymbol{,Z}}_{\boldsymbol{1:j-1}} \right]\boldsymbol{P}\left[ {\vec{\boldsymbol{X}}}_{\boldsymbol{j}}\boldsymbol{|}\boldsymbol{Z}_{\boldsymbol{1:j-1}} \right]}{\boldsymbol{P}\left[ \boldsymbol{Z}_{\boldsymbol{j}}\boldsymbol{|}\boldsymbol{Z}_{\boldsymbol{1:j-1}} \right]}\boldsymbol{,} \propto\boldsymbol{P}\left[ \boldsymbol{Z}_{\boldsymbol{j}}\boldsymbol{|}{\vec{\boldsymbol{X}}}_{\boldsymbol{j}} \right]\boldsymbol{P}\left[ {\vec{\boldsymbol{X}}}_{\boldsymbol{j}}\boldsymbol{|}\boldsymbol{Z}_{\boldsymbol{1:j-1}} \right]$$

The filtered distribution is approximated by the Gaussian distribution using a Taylor expansion of its logarithm up to the second-order term (Laplace Approximation). The mean and covariance of the filtered distribution $\vec{x}_{j|j}$, $\Sigma_{j|j}$ are given as follows:

$$\frac{\boldsymbol{d}}{\boldsymbol{d}{\vec{\boldsymbol{x}}}_{\boldsymbol{j}}}\mathbf{log}\boldsymbol{p}\left( {\vec{\boldsymbol{x}}}_{\boldsymbol{j}}\boldsymbol{|}\boldsymbol{Z}_{\boldsymbol{1:j}} \right)\boldsymbol{|}_{\vec{x}_{j|j}}\boldsymbol{=0,}(24)$$

$$\boldsymbol{\Sigma}_{\boldsymbol{j|j}}^{\boldsymbol{-1}}\boldsymbol{=-H}\left( \mathbf{log}\boldsymbol{p}\left( {\vec{\boldsymbol{x}}}_{\boldsymbol{j}}\boldsymbol{|}\boldsymbol{Z}_{\boldsymbol{1:j}} \right) \right)\boldsymbol{|}_{\vec{x}_{j|j}}\boldsymbol{,}(25)$$

where $\boldsymbol{H(f)}$ is the Hessian matrix of f, i.e., $\boldsymbol{H}\left( \mathbf{f} \right)\boldsymbol{=}\frac{\boldsymbol{d}^{\boldsymbol{2}}}{\boldsymbol{d}{{\vec{\boldsymbol{x}}}_{\boldsymbol{j}}}^{\boldsymbol{2}}}\boldsymbol{f}$. The Bayesian estimators at the final time step, $\vec{x}_{N-1|N-1}$, is calculated by iterating the prediction and filtering algorithms (22-25) from the initial value $\vec{x}_{0|0}$, $\Sigma_{0|0}$.

**Smoothing Algorithm**

The mean and covariance of the smoothed distribution $\vec{x}_{j|N-1}$, $\Sigma_{j|N-1}$ are given as follows:

$${\vec{\boldsymbol{x}}}_{\boldsymbol{j|N-1}}\boldsymbol{=}{\vec{\boldsymbol{x}}}_{\boldsymbol{j|j}}\boldsymbol{+}\boldsymbol{A}_{\boldsymbol{j}}\left( {\vec{\boldsymbol{x}}}_{\boldsymbol{j+1|N-1}}\boldsymbol{-}{\vec{\boldsymbol{x}}}_{\boldsymbol{j+1|j}} \right)\boldsymbol{,} (26)$$

$$\boldsymbol{\Sigma}_{\boldsymbol{j|N-1}}\boldsymbol{=}\boldsymbol{\Sigma}_{\boldsymbol{j|j}}\boldsymbol{+}\boldsymbol{A}_{\boldsymbol{j}}\left( \boldsymbol{\Sigma}_{\boldsymbol{j+1|N-1}}\boldsymbol{-}\boldsymbol{\Sigma}_{\boldsymbol{j+1|j}} \right)\boldsymbol{A}_{\boldsymbol{j}}^{\boldsymbol{T}}\boldsymbol{,} (27)$$

where

$$\boldsymbol{A}_{\boldsymbol{j}}\boldsymbol{=}{\boldsymbol{\Sigma}_{\boldsymbol{j|j}}\boldsymbol{\Sigma}}_{\boldsymbol{j+1|j}}^{\boldsymbol{-1}}\boldsymbol{,} (28)$$

The Bayesian estimators, $\vec{x}_{j|N-1} \left( j=1,\cdots,N-1 \right)$, are calculated by iterating the smoothing algorithm (26, 27) from the initial value $\vec{x}_{N-1|N-1}$, $\Sigma_{N-1|N-1}$.

**Covariance Algorithm**

The covariance algorithm used to calculate the conditional covariance $\boldsymbol{\Sigma}_{\boldsymbol{j+1, j|N-1}}$ is given as follows:

$$\boldsymbol{\Sigma}_{\boldsymbol{j+1, j|N-1}}\boldsymbol{=}\mathbf{A}_{\boldsymbol{j}}\boldsymbol{\Sigma}_{\boldsymbol{j+1|N-1}}\boldsymbol{,} (29)$$

The iterative formulae of the EM algorithm (17, 18) is rewritten as follows:

$$\boldsymbol{E}\left[ \left( \boldsymbol{M}_{\boldsymbol{j+1}}\boldsymbol{-}\boldsymbol{M}_{\boldsymbol{j}} \right)^{\boldsymbol{2}}\boldsymbol{|}\boldsymbol{Z}_{\boldsymbol{1:N-1}}\boldsymbol{;}{\vec{\boldsymbol{\theta}}}_{\boldsymbol{k}} \right]\boldsymbol{=(}\boldsymbol{x}_{\boldsymbol{j+1|N-1}}^{\left( \boldsymbol{1} \right)}{\boldsymbol{-}\boldsymbol{x}_{\boldsymbol{j|N-1}}^{\left( \boldsymbol{1} \right)}\boldsymbol{)}}^{\boldsymbol{2}}$$

$$\boldsymbol{+}\boldsymbol{\Sigma}_{\boldsymbol{j+1|N-1}}^{\boldsymbol{(1,1)}}\boldsymbol{+}\boldsymbol{\Sigma}_{\boldsymbol{j|N-1}}^{\boldsymbol{(1,1)}}\boldsymbol{-2}\boldsymbol{\Sigma}_{\boldsymbol{j+1,j|N-1}}^{\left( \boldsymbol{1,1} \right)}\boldsymbol{,}(30)$$

$$\boldsymbol{E}\left[ \left( \boldsymbol{S}_{\boldsymbol{j+1}}\boldsymbol{-}\boldsymbol{S}_{\boldsymbol{j}} \right)^{\boldsymbol{2}}\boldsymbol{|}\boldsymbol{Z}_{\boldsymbol{1:N-1}}\boldsymbol{;}{\vec{\boldsymbol{\theta}}}_{\boldsymbol{k}} \right]\boldsymbol{=(}\boldsymbol{x}_{\boldsymbol{j+1|N-1}}^{\left( \boldsymbol{2} \right)}{\boldsymbol{-}\boldsymbol{x}_{\boldsymbol{j|N-1}}^{\left( \boldsymbol{2} \right)}\boldsymbol{)}}^{\boldsymbol{2}}$$

$$\boldsymbol{+}\boldsymbol{\Sigma}_{\boldsymbol{j+1|N-1}}^{\boldsymbol{(2,2)}}\boldsymbol{+}\boldsymbol{\Sigma}_{\boldsymbol{j|N-1}}^{\boldsymbol{(2,2)}}\boldsymbol{-2}\boldsymbol{\Sigma}_{\boldsymbol{j+1,j|N-1}}^{\left( \boldsymbol{2,2} \right)}\boldsymbol{,}(31)$$

where $\boldsymbol{x}_{\boldsymbol{j|N-1}}^{\left( \boldsymbol{k} \right)}$ is the k-th component of the vector $\vec{x}_{j|N-1}$, $\boldsymbol{\Sigma}_{\boldsymbol{j|N-1}}^{\boldsymbol{(k,l)}}$ is the $\boldsymbol{(k,l)}$ component of the matrix $\boldsymbol{\Sigma}_{\boldsymbol{j|N-1}}$ and $\boldsymbol{\Sigma}_{\boldsymbol{j+1,j|N-1}}^{\boldsymbol{(k,l)}}$ is the $\boldsymbol{(k,l)}$ component of the matrix $\boldsymbol{\Sigma}_{\boldsymbol{j+1,j|N-1}}$.
